# Detection of Nitrate-Reducing/Denitrifying Bacteria from Contaminated and Uncontaminated Tallgrass Prairie Soil: Limitations of PCR Primers

**DOI:** 10.3390/microorganisms12101981

**Published:** 2024-09-30

**Authors:** Samer M. AbuBakr, Fares Z. Najar, Kathleen E. Duncan

**Affiliations:** 1 Biology Program, School of Integrated Sciences, Sustainability, and Public Health, College of Health, Science, and Technology, University of Illinois at Springfield, Springfield, IL 62703, USA; 2 High Performance Computing Center, Division of the Vice President for Research, Oklahoma State University, Stillwater, OK 74078, USA; fnajar@okstate.edu; 3 School of Biological Sciences, University of Oklahoma, Norman, OK 73019, USA; kathleen.e.duncan-1@ou.edu

**Keywords:** nitrate-reducing bacteria, denitrifying bacteria, oil/brine contaminated soils, PCR

## Abstract

Contamination of soil by spills of crude oil and oilfield brine is known to affect the species composition and functioning of soil microbial communities. However, the effect of such contamination on nitrogen cycling, an important biogeochemical cycle in tallgrass prairie soil, is less well known. Detecting nitrate-reducing (NR) and denitrifying (DN) bacteria via PCR amplification of the genes essential for these processes depends on how well PCR primers match the sequences of these bacteria. In this study, we enriched for NR and DN bacteria from oil/brine tallgrass prairie soil contaminated 5–10 years previously versus those cultured from uncontaminated soil, confirmed the capacity of 75 strains isolated from the enrichments to reduce nitrate and/nitrite, then screened the strains with primers specific to seven nitrogen cycle functional genes. The strains comprised a phylogenetically diverse group of NR and DN bacteria, with proportionately more γ-Proteobacteria in oil-contaminated sites and more Bacilli in brine-contaminated sites, suggesting some residual effect of the contaminants on the NR and DN species distribution. Around 82% of the strains shown to reduce nitrate/nitrite would not be identified as NR and DN bacteria by the battery of NR and DN primers used. Our results indicate an urgent need to expand the NR/DN functional gene primer database by first identifying novel NR/DN strains through their capacity to reduce nitrate/nitrite.

## 1. Introduction

Soil is a very complex environment with a high diversity of bacteria [1]. After contamination of soil with crude oil and/or oilfield brine (e.g., water containing salts in solution, produced along with oil), certain groups of bacteria come to dominate the contaminated sites, particularly α-, β-, γ-, and δ Proteobacteria in the crude oil contaminated sites [2,3,4] and Firmicutes [5] and some *Pseudomonas* species [6] in brine contaminated sites. The alteration in bacterial species composition and numbers might be expected to affect biogeochemical cycling in soils. In tallgrass prairie soils, nitrogen is a limiting nutrient for the growth of plants and soil decomposition rates [7]. Therefore, the effect of oil/brine contamination on nitrogen-cycling bacteria is of particular concern in tallgrass prairie soil. In fact, nitrate-reducing (NR) and denitrifying (DN) bacteria are found in oil/brine contaminated soils [8,9,10,11], including Tallgrass Prairie Preserve (TPP) soil contaminated by oil/brine [12,13], but a detailed examination of their species composition and abundance in oil/brine contaminated TPP soil is unknown. In particular, the effects of long-term residual oil and brine contamination on NR and DN species composition are not known.

Nitrate-reducing microorganisms represent a widespread group with members among α-, β-, and γ-Proteobacteria, some members of Firmicutes, and even *Archaea*. These microorganisms use nitrate as an alternative electron acceptor that makes them able to obtain energy from dissimilatory reduction of nitrate into nitrite by nitrate reductase enzymes [14]. Nitrite can be reduced into gaseous nitrogen compounds by denitrification or by dissimilatory nitrate reduction into ammonia (DNRA) [15,16]. Nitrate reduction to nitrite is catalyzed by two different types of nitrate reductases, either membrane-bound encoded by the *narGHJI* operon or periplasmic encoded by the *napABC* operon [17]. Some NR bacteria can harbor either *narG*, *napA*, or both [18,19].

The denitrification process consists of four reaction steps in which nitrate is reduced, and nitrogen is lost in the form of nitrogenous gasses [20]. Because nitrite reductase produces NO, the first gaseous product, nitrite reductase is a significant enzyme in the denitrification pathway [21,22] and signals the potential for loss of nitrogen from the soil ecosystem. Nitrite reductase is found in two different forms. The first form contains copper and is encoded by *nirK*, while the second contains cytochromes (hemes) *c* and *d_1_* and is encoded by *nirS* [14,22,23,24,25].

The reduction of nitric oxide is catalyzed by nitric oxide reductase, whose small and large subunits are encoded by *norC* and *norB*, respectively [26]. Based on the European Molecular Biology Laboratory (EMBL) sequence database, norB sequences are grouped in two very distinct branches where the first class encodes the cytochrome bc-type complex (cNorB) while the second class encodes the quinol-oxidizing single-subunit class (qNorB) [26]. The last step in the denitrification pathway is the reduction of nitrous oxide to N_2,_ which is catalyzed by nitrous oxide reductase encoded by the *nosZ* gene.

Since denitrification also is common among phylogenetically unrelated groups, it is very unsuitable to investigate communities of DN bacteria by using an approach based on 16S rRNA gene sequences [23,27]. Therefore, genes coding for such key steps as nitrite and nitrous oxide reduction are often used to detect DN bacteria [23,28,29,30,31,32].

Nitrate reducers and denitrifiers are an excellent model system for investigating the response of an important component of the N-cycling soil community to the contaminants of crude oil and brine as they contain members from among many different groups of microorganisms. The objectives of this study were (1) to determine if the species composition of culturable dissimilatory nitrate reducers and denitrifiers in contaminated and uncontaminated soils were as predicted by their tolerance to and/or utilization of the particular contaminants and (2) evaluate whether dissimilatory nitrate-reducing and/or denitrifying bacteria can be more reliably identified using PCR primers specific for a broad suite of NR and DN genes versus screening for the capacity to reduce nitrate and/or nitrite. The PCR products were sequenced in order to determine that the expected genes were amplified.

The sites used in this study were located within the Tallgrass Prairie Preserve (TPP) in Osage County, Oklahoma. Mineral rights are owned by the Osage Nation, and oil, together with produced water (brine), has been produced for over 100 years [33]. Accidental spills of oil and/or brine have occurred, and our research on the most effective means of remediation/restoration has been described in detail in previous publications [12,13,33,34,35,36,37].

In this study, bacterial strains were isolated from crude oil- and oil/brine-contaminated and uncontaminated tallgrass prairie soils from TPP near Pawhuska, Oklahoma. Five to 10 years had lapsed between the contamination event and soil collection for isolation of the bacterial strains, yet somewhat elevated levels of petroleum hydrocarbons, Na^+^, Cl^−^, and NO_3_^−^ persisted [13]. The nature of the contaminant was hypothesized to alter the species composition of NR and DN bacteria in certain ways, e.g., we hypothesized that NR and DN bacteria in the crude oil-contaminated sites would contain a greater proportion of strains affiliated with groups, such as γ-Proteobacteria, known to increase in crude-oil contaminated sites [38,39]. Some strains of γ-Proteobacteria are noted for the ability to use crude oil components as sole carbon sources [40,41], although the ability to degrade petroleum compounds is also found in many other microbial groups [42,43]. Since high levels of sodium chloride inhibit certain groups of bacteria [44,45], although Alcántara-Hernández et al. [46] found *napA* and *narG* genes in highly saline soil, we hypothesized that the NR and DN bacteria from oil/brine contaminated sites would be biased towards those groups that are salt tolerant, such as *Bacillus* [5] and *Pseudomonas* [6,47]. Our strains represent some of the diversity of readily culturable NR/DN bacteria present at these sites, but we acknowledge that this is not an exhaustive survey.

## 2. Materials and Methods

### 2.1. Summary of Sampling Sites and Sampling Protocol

The sites used in this study were located in the TPP in Osage County, Oklahoma. The bacterial strains described in this study were obtained from soil samples collected from the following sites 5–10 years post-contamination (see AbuBakr et al., 2019 [13]) for contaminant values when sampled for the current study): two sites (G5, G7) contaminated by both crude oil and brine (salt water), and three sites contaminated by crude oil (J6-F, J6-NF, LF). Samples from four uncontaminated sites adjacent to the contaminated sites (G5P, J6P; G7P, LFP) were collected at the same time as for contaminated sites. Both contaminants were present in sites G5 and G7, with a brine/oil ratio of approximately 10:1 (vol/vol) [36]. In brief, a trowel was used to remove five shallow scoops of soil from five widely spaced points within a 25 m radius from approximately the top four inches of soil. Samples were initially mixed in Whirl-pac^®^ bags in the field using autoclaved spoons and, later in the lab, were homogenized in autoclaved beakers by mixing with autoclaved spoons.

### 2.2. Selection and Isolation of Nitrate- and Nitrite-Reducing Bacteria

The homogenized soils were serially diluted at a 1:10 ratio (2 g of soil/18 mL saline) up to a final dilution of 10^−8^ in sterile isotonic (0.85%) saline. Twenty microliters of each dilution were used to inoculate 180 µL of the nitrate broth in a 5-fold replicate in a microtiter plate. Nitrate broth medium consisted of 2.5 g of NaNO_3_ added to 500 mL of nutrient broth (Difco Inc., Detroit, MI, USA) before autoclaving. Nitrate broth is used to enrich bacteria that reduce nitrate into nitrite or produce N_2_ gas [48]. The antifungal agent, cycloheximide (100 µg/mL final concentration) (Sigma-Aldrich Corporation, St. Louis, MO, USA), was added after autoclaving. Internal controls for replicability included cultures of strains positive for NR (*Stenotrophomonas maltophilia* ATCC 13637), DN (*Pseudomonas aeruginosa* ATCC 27853), or negative for NR or DN (*Pseudomonas putida* pG7) grown in nitrate broth were added singly to three microtiter plate wells to serve as positive or negative controls for DN/NR. Additionally, three uninoculated wells were included as controls for contamination. After two weeks of incubation at room temperature (23–25 °C) in containers that excluded light [49], all wells were examined for evidence of growth (turbidity), and those that were turbid were scored positive for growth of aerobic/facultative anaerobic heterotrophic bacteria. Subsequently, half of the volume of the wells was transferred to a fresh set of microtiter plates containing nitrate broth and tested for the presence of the expected products of nitrate reduction or denitrification using Griess reagents [50,51]. Briefly, two drops of reagent 1 (Sulfanilic acid) and two drops of reagent 2 (N, N-Dimethyl-1- naphthylamine) (bioMérieux Vitek, Inc., Hazelwood, MO, USA) were added to every well. The presence of nitrite is detected by a red color after the addition of reagents 1 and 2, e.g., indicating nitrate reduction. If the medium remained colorless, e.g., no nitrite formed, it is due either to lack reduction of nitrate, e.g., no nitrate reduction, or the reduction of nitrite to other products, such as NO, N_2_O, or N_2_, hence denitrification. If the medium remained colorless, zinc metal dust (Mallinckrodt Chemical Works, St. Louis, MO, USA) was used to detect if unreduced nitrate was present by reducing nitrate to nitrite and turning the medium pink or red. Therefore, pink or red medium color after zinc dust indicates nitrate was not reduced. Therefore, neither NR nor DN bacteria were present, or if present, did not reduce nitrate under the test conditions. If colorless after zinc dust, the well was assumed to contain DN bacteria. However, since only the loss of nitrate and nitrite was demonstrated, bacteria in the colorless wells could be incomplete denitrifiers rather than performing the entire denitrification pathway, as this protocol does not screen directly for gaseous nitrogen compounds that are products of later steps in the denitrification pathway nor for ammonium produced by DNRA.

### 2.3. Obtaining Pure Cultures from Microtiter Plate Wells Positive for DN or NR

Nutrient agar plates (Difco Inc., Detroit, MI, USA) were quadrant streaked with 25 µL from the original set of microtiter plates, chosen randomly from 10^−2^ to 10^−5^ dilutions that scored positive for growth and nitrate reduction or denitrification. Positive results for nitrate and nitrite reduction were commonly obtained from the 10^−2^ to 10^−7^ dilution wells. The nutrient agar plates were incubated at 30 °C. A colony chosen from a plate was streaked at least three times in succession from a single colony to a fresh plate to obtain pure cultures. Seventy-five strains in total were isolated from microtiter plate wells for further testing. Each purified strain was retested for the ability to reduce nitrate or nitrite using the microtiter plate format, control strains, and Griess reagents as described previously.

### 2.4. DNA Extraction from Strains

Genomic DNA was isolated from cells using a bead-beating method. Each of the 75 strains was streaked on a nutrient agar plate and incubated for 1–3 days at 30 °C until good colony growth was observed. Then, a single colony from each plate was used to inoculate 5 mL nutrient broth and incubated at 30 °C until turbid growth was seen. One to 2 mL culture was centrifuged at 6000xg for 10 min to provide a pellet size of approximately 10^9^ bacteria. The FastDNA^®^SPIN Kit (QBIOgene, Solon, OH, USA) was used to extract DNA from the cells by following the manufacturer’s directions. Extracted DNA was stored at −20 °C. Controls to detect contamination during DNA extraction included processing one kit unit to which sterile water rather than cells was added.

### 2.5. Molecular Detection of 16S rRNA Gene Sequences and Nitrate Reduction/Denitrification Functional Genes

Determination of the 16S rRNA gene sequence and screening for nitrate reductase genes (e.g., *napA* and *narG*) and nitrite reductase genes (e.g., *nirK* and *nirS*) were performed for all 75 strains. The 16S rRNA gene sequence was amplified using primer pairs 27F/1492R [52]. Fragments from nitrate reductase and nitrite reductase genes were amplified using specific primer pairs as listed in Supplemental Table S1. Thirty-three strains that were shown to reduce nitrate and/or nitrite were chosen as representative of the NR and DN 16S rRNA phylogenetic diversity and screened for the presence of *qnorB*, *cnorB*, and *nosZ* functional denitrifying genes (specific primers listed in Supplemental Table S1). Five strains were obtained from ATCC for use as positive controls for PCR detection of denitrification functional genes (Supplemental Table S1). The PCR products were sequenced to confirm their matching to the expected genes.

### 2.6. DNA Sequencing and Analysis

PCR products were prepared for sequencing using ExoSAP-IT (USBiochemicals, Cleveland, OH, USA). The nucleotide sequences of the PCR products were determined at the Oklahoma Medical Research Foundation (OMRF) in Oklahoma City, Oklahoma. Sequencher (Windows version 4.2; Gene Codes Corp., Ann Arbor, MI, USA) was used to analyze the chromatograms and produce a consensus sequence. The sequence of the 16S rRNA gene and nitrate reduction/denitrification functional genes were compared to the GenBank database using BLASTN searches (Basic Local Alignment Search Tool) [53]. The taxonomic assignation of the 16S rRNA gene sequence was determined by the Naïve Bayesian rRNA Classifier of the Ribosomal Database Project II (RDP) [54]. The 16S rRNA sequences were deposited in GenBank under accession numbers JQ917765-JQ917839. Sequences confirmed as genes of nitrate reduction/denitrification were deposited in GenBank under accession numbers OR416960-OR416973.

16S rRNA gene sequences of the 75 strains were clustered as operational taxonomic units (OTUs) at an overlap identity cut-off of 98% by MOTHUR software ver1.23 [55]. Unique sequences were aligned against the SILVA reference alignment database [56] using the NAST-aligner [57] and subsequently screened for chimeras using UChime [58]. A distance matrix was generated and used to cluster sequences into OTUs at 98% similarity. A representative sequence from each OTU was assigned a taxonomic classification based on the naïve Bayesian classifier [54]. The representative OTUs, together with closely related sequences from the GenBank database, were aligned by Clustal-X software version 1.81 [59]. As implemented in CLUSTALX, the neighbor-joining method of Saitou and Nei (1987) [60] was used to construct the phylogenetic tree among the aligned sequences. Distances were calculated as % divergence between all pairs of sequences from the multiple alignments, alignment positions with gaps were excluded from the analysis, and the Kimura two-parameter distance correction for multiple substitutions was applied [61]. The support for the tree branches was estimated from 1000 bootstrap replicates [62]. Only bootstrap values greater than 700 are shown on the phylogenetic trees.

## 3. Results

### 3.1. Distribution of Strains by Origin and Capacity to Reduce Nitrate/Nitrite

**Figure 1:**
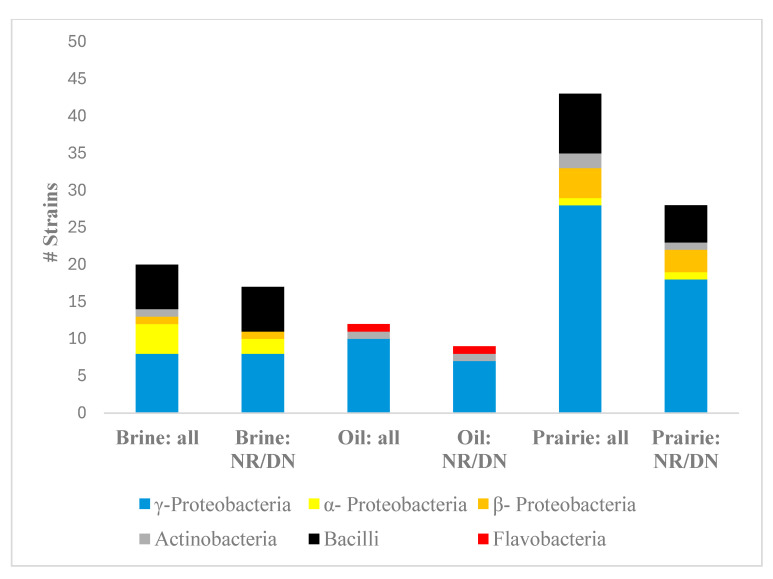
Taxonomic Affiliation of Strains. The number of strains affiliated with different higher taxonomic level bacterial groups found in oil-, brine-, and prairie-soils. The 16S rRNA gene sequences of seventy-five strains were classified by the RDP Classifier tool. Brine: isolated from sites contaminated primarily by oil field brine but also by crude oil (10:1 vol/vol), G5 and G7. Oil: isolated from sites contaminated by crude oil, J6-F, J6-NF, and LF. Prairie: isolated from uncontaminated sites, G5P, G7P, J6P, and LFP. “All” indicates the distribution of the 75 strains isolated after enrichment in nitrate broth, and “NR/DN” indicates the distribution of 54 strains confirmed as nitrate/nitrite reducers after incubation in nitrate broth as described in Materials and Methods.

Seventy-five strains were isolated from tallgrass prairie soils after enrichment in nitrate broth (Supplemental Table S2). Out of the 75 strains tested, 48 strains were identified as nitrate reducers, six as nitrite reducers, and 21 strains did not show the ability to reduce nitrate or nitrite under the test conditions (Table 1). Among the 54 that showed a reduction of nitrate/nitrite, 17 were obtained from brine/oil-contaminated soils, nine from oil-contaminated soils, and 28 from uncontaminated prairie soils (Table 1, Figure 1). The highest proportion of nitrate/nitrite reducers isolated from a particular type of site was obtained from brine/oil contaminated soil (85%), next highest from oil-contaminated sites (75%), and the lowest proportion from the uncontaminated sites (65%).

The microtiter plate test conditions using Griess reagents did correctly identify nitrate-reducing control strains as nitrate reducers, while neither nitrate nor nitrite was detected after the growth of control strain denitrifiers. The Griess protocol does not screen for gaseous nitrogen compounds that are products of later steps in the denitrification pathway. Therefore, for the purpose of this study, strains that reduced both nitrate and nitrite are designated as denitrifiers (DN), with the understanding that strains labeled as “DN” may either be bacteria capable of reducing nitrate to nitrite and nitrite to gaseous nitrogen compounds that are not dinitrogen (e.g., incomplete denitrification) or “true” denitrifying bacteria, the latter capable of reducing nitrate completely to dinitrogen (“complete denitrification”).

### 3.2. 16S rRNA Phylogenetic Affiliations of the Strains

16S rRNA gene sequences were used to classify the 75 strains. The 75 strains consisted of 61% γ-Proteobacteria, 7% α-Proteobacteria, 7% β-Proteobacteria, 19% Bacilli, 5% Actinobacteria, and 1% Flavobacteria (Table 1). However, since strains were isolated from wells representing different dilutions (Supplemental Table S2), it is assumed that strains originating from the 10^−4^ or 10^−5^ wells represented species that were more abundant than those isolated solely from the 10^−2^ wells. Based on their origin from 10^−4^ or 10^−5^ wells, the most abundant bacteria were γ-Proteobacteria (59%), α-Proteobacteria (23%), Actinobacteria (12%), and Bacilli (1%). These abundance results corrected for the dilution show that γ-Proteobacteria were still the major group, but there were likely more α-Proteobacteria and fewer Bacilli than calculated from a direct count of the number of isolates (Supplemental Table S2). Note that due to isolating strains from different dilutions and small sample sizes, % values are descriptive and not statistically valid differences.

γ-Proteobacteria were represented by 46 strains (Table 1, Figure 1). The two most abundant genera of γ-Proteobacteria were *Stenotrophomonas* (27 strains) and *Pseudomonas* (9 strains, Table 1). The two most abundant genera of Bacilli were *Brevibacillus* (8 strains) and *Bacillus* (3 strains). Table 2 also shows that some genera (e.g., *Aeromonas*, *Serratia*, *Enterobacter*, *Ensifer*, *Bosea*, *Brevundimonas*, *Arthrobacter*, *Microbacterium*, *Paenibacillus*, and *Chryseobacterium*) were isolated from the contaminated sites, but not from the prairie sites. However, most of these genera were represented by single strains.

**Figure 2:**
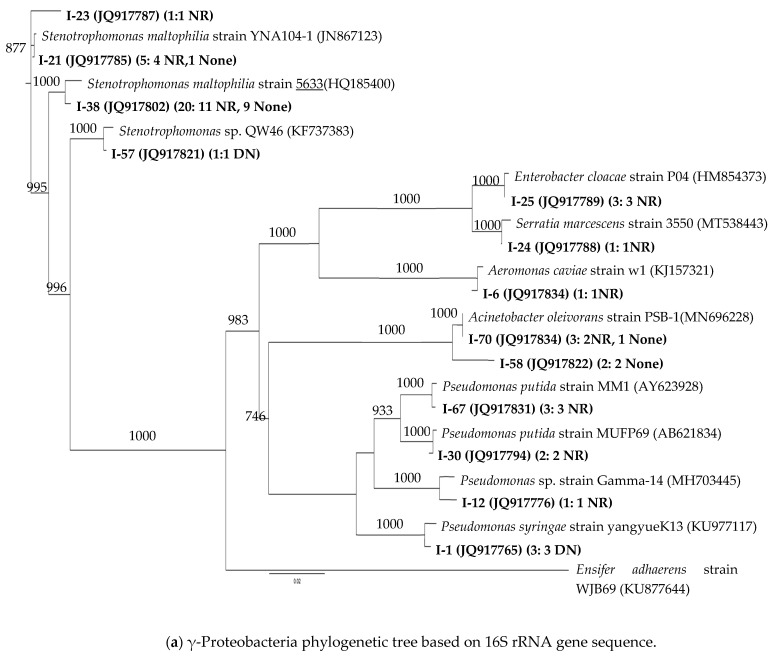

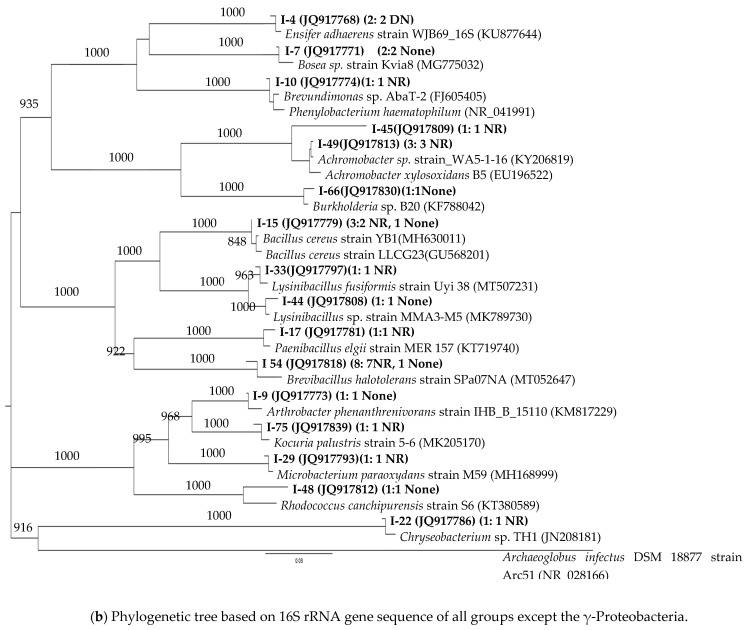
Phylogenetic tree of the 16S rRNA gene of 75 bacterial strains isolated from tallgrass prairie soils with respect to related sequences. There were 29 OTUs (indicated in bold letters) at the 98% level of similarity. One strain representing each OTU was used for phylogenetic analysis. The number of strains contained in each OTU is included between parentheses next to the representative strain. The numbers of strains of NR (nitrate-reducing) bacteria, DN (nitrite-reducing) bacteria, and None (strains that did not reduce nitrate or nitrite) are also included next to the total number of strains included in the OTU. The tree is constructed from approximately 1400 bp 16S rRNA gene sequence using the neighbor-joining algorithm. One thousand bootstrap replications were performed; only values greater than 700 are shown. (**a**) (Bar: 0.02 nucleotide substitutions per nucleotide) shows the γ-Proteobacteria where the sequence of *Ensifer adhaerens* strain WJB69 (KU877644) was included as the outgroup, while (**b**) (Bar: 0.03 nucleotide substitutions per nucleotide) shows the remaining groups where the sequence of *Archaeoglobus infectus* DSM 18877 strain Arc51 (NR_028166) was included as the outgroup.

Most strains had 16S rRNA gene sequences highly similar (98–100%) to those of previously described isolates. The 75 strains were grouped into 29 OTUs based on 98% 16S rRNA sequence similarity. Figure 2a,b shows the phylogenetic affiliation of a representative sequence from each OTU, the total number of sequences in the OTU, and the NR and DN physiology of the strains in the OTU. Note that even grouping strains at the 98% level of 16S rRNA gene sequence similarity did not always create OTUs in which all strains had the same NR and DN physiology.

Figure 2a shows the phylogenetic affiliation of the γ-Proteobacteria. The nine *Pseudomonas* strains were grouped in four OTUs, each of which grouped together strains with the same nitrate reduction physiology. The three *Pseudomonas* strains represented by strain I-1 that reduced nitrite after incubation in nitrate broth in the microtiter plate (e.g., DN) were members of the same OTU, most similar to the 16S rRNA sequence of *Pseudomonas syringae* strain yangyueK13 (KU977117) while the other six *Pseudomonas* strains were nitrate reducers (e.g., NR) with five of the six most similar to *P. putida* strains. Other OTUs comprised of multiple strains that had the same nitrate reduction physiology were those most similar to *Enterobacter cloacae* (3NR) and *Acinetobacter oleivorans* (2 “None”, did not reduce nitrate, e.g., neither NR nor DN). However, a second OTU closest to *Acinetobacter oleivorans* consisted of a group of three strains, two of which were nitrate reducers and one that did not reduce nitrate or nitrite. The single strains of *Aeromonas* and *Serratia* were NR.

The 27 *Stenotrophomonas* strains were split into four OTUs, with variation in nitrate reduction physiology within the OTUs containing more than one strain. Twenty out of the 27 *Stenotrophomonas* strains clustered into one OTU, with the closest 16S rRNA gene sequence match to *S. maltophilia* strain 5633 with 11 nitrate reducers and nine that did not reduce nitrate or nitrite. A second *Stenotrophomonas* OTU, which matched most closely to *S. maltophilia* strain YNA104-1, had five strains (e.g., four nitrate reducers, and one did not reduce nitrate or nitrite). One strain, I-23, was grouped most closely with *S. maltophilia* strain YNA104-1 but distinct from the previous OTU. On the other hand, the only *Stenotrophomonas* (e.g., I-57, JQ917821) that reduced nitrite after incubation in nitrate broth (e.g., DN) was clustered by itself with 99.49% 16S rRNA gene sequence similarity to *Stenotrophomonas* sp. QW46 (KF737383).

Figure 2b shows the remaining taxa. Some strains appeared very similar in both 16S rRNA sequence and their ability to reduce nitrate/nitrite. For the most part, strains grouped together by their similarity in 16S rRNA gene sequence as belonging to the same OTU had the same nitrate/nitrite reduction phenotype. All four *Achromobacter* strains, although split into twp OTUs, were nitrate reducers, as were two strains most similar to *Ensifer adhaerens*. Neither of the two strains most similar to *Bosea* reduced nitrate, although some species of *Bosea* are denitrifiers. However, all eight *Brevibacillus* strains were grouped together as one OTU even though seven were nitrate reducers, and one did not reduce nitrate or nitrite. In addition, all three *Bacillus* strains (two nitrate reducers and one did not reduce nitrate or nitrite) were grouped together with *Bacillus cereus* strains. The two *Lysinibacillus* strains (one nitrate reducer and one did not reduce nitrate or nitrite) were most similar to different species of *Lysinibacillus*. We obtained single strains of the following genera (Table 1): *Phenylobacterium* (isolate #10) (nitrate reducer), which is 99.93% similar to *Brevundimonas* sp. AbaT-2 (FJ605405) (Figure 2a), *Burkholderia* (did not reduce nitrate or nitrite), *Arthrobacter* (did not reduce nitrate or nitrite), *Rhodococcus* (did not reduce nitrate or nitrite), *Microbacterium* (nitrate reducer), *Kocuria* (nitrate reducer), *Paenibacillus* (nitrate reducer), and *Chryseobacterium* (nitrate reducer).

### 3.3. Molecular Detection of Nitrate-Reducing Functional Genes napA and narG

For all functional genes, DNA sequencing of the PCR product was performed and matched against sequences in GenBank in order to confirm that the sequence of the PCR product matched that of the expected gene for its full length. Many strains that were shown to reduce nitrate and/or nitrite were not amplified by the primers for functional genes. In some cases, PCR products were produced but failed due to reading frame shifts that produced stop codons and/or contained regions that had little to no similarity to the expected gene. Gene sequences of control strains, by contrast, did not have these problems, nor did the 16S rRNA sequences of any strain. Supplemental Table S3 lists for each strain both any detected PCR products (“**+**”) and those verified as the correct sequence (“**++**”). Only the verified sequences are used for the following analyses.

Primers designed to detect *napA* and *narG* nitrate reductase genes were used to screen for the presence of nitrate-reducing genes in all 75 strains. The *napA* nucleotide sequence was obtained from seven strains out of the 75 screened (Table 2 and Table 3). The seven strains include α-Proteobacteria (three strains: two *Bosea* and one *Ensifer*), β-Proteobacteria (one strain: *Achromobacter*), and γ-Proteobacteria (three strains: *Pseudomonas*). The match similarity between the *napA* sequences of these seven strains (e.g., OR416962-OR416968) and *napA* sequences of other strains from GenBank ranged from 91% to 98% (Supplemental Table S4). Gene sequences are highly similar (99.61%) to that coding for membrane-bound nitrate reductase (*narG*) of *Stenotrophomonas geniculata* strain BR23 (CP134450) were confirmed in two *Stenotrophomonas* strains (e.g., strains I-39, OR416969 and I-42, OR416970) (Supplemental Table S4).

### 3.4. Molecular Detection of Denitrifying Functional Genes nirS, nirK, cnorB, qnorB, and nosZ

All 75 strains were screened for the presence of nitrite reductase gene(s) using primers designed to detect the *nirS* nitrite reductase gene and *nirK* nitrite reductase gene. In addition, screening with c*norB*, *qnorB*, and *nosZ* primers, as another indication of denitrification genes, was applied to a subset of the chosen 33 strains that showed the ability to reduce nitrate/nitrite in the microtiter plate assay and that represented the phylogenetic diversity of the strains. The selected 33 strains are indicated in bold letters in Supplemental Table S3 and summarized in Table 2.

A total of four strains were found, where one or more denitrification functional genes were detected by PCR amplification and confirmed by sequencing to have high similarity to the denitrification genes (Table 3). The gene sequence coding for nitrite reductase (*nirS*) was detected in two *Pseudomonas* strains (I-1 and I-2, Supplemental Table S4), isolated from oil/brine-contaminated soil, but not in a third *Pseudomonas* strain classified as DN. *Pseudomonas frederiksbergensis* strain AS1 provided the best match to the *nirS* of I-1 and I-2, as well as to I-1 *napA*. Strain AS1 was listed as a naphthalene-degrader (CP018319), while the description of the type strain of *P. frederiksbergensis* notes it is a denitrifier, degraded phenanthrene and was isolated from soil contaminated by coal gasification. No *nirK* sequence gene was confirmed in any of the 33 strains screened for this gene using primers specific for *nirK* (Supplemental Tables S1 and S3).

The gene coding for nitric oxide reductase (c*norB*) was confirmed in *Ensifer* strain I-4 (OR416960) and *Pseudomonas* strain I-65 (OR416961) (Table 3, Supplemental Table S4). The highest % match (96%) for I-4 *cnorB* was to *E. adhaerens* strain NER9 (CP101518), isolated from the rhizosphere of wheat grown in cadmium-polluted soil [63], but the strain was not examined for the ability to denitrify. The highest % match (89.21%) for the I-65 *cnorB* was to Pseudomonadaceae bacterium isolate f13e6c2e-50f2-4c75-b4f3-64e691971528 (OY763427). No *qnorB* sequence was detected in any of the 33 strains screened for this gene using primers specific for *qnorB* (Supplemental Table S1).

Primers specific for a portion of the nitrous oxide reductase gene were successful in amplifying the *nosZ* fragment from *Pseudomonas* strain I-65 (OR416973, Table 3, Supplemental Table S4). Its highest % match (97.35%) was to a region identified as *nosZ* in the genome sequence of *Pseudomonas* sp. P9_35 (CP125373), but P9_35 was not tested for the ability to denitrify.

## 4. Discussion

This study investigates the diversity of NR and DN bacteria isolated from tallgrass prairie soils and their dissimilatory nitrate reduction/denitrification pathway genes. Although sample sizes were small, nitrate-reducing/denitrifying bacteria were readily isolated from oil and/or brine/oil-contaminated soils after enrichment in nitrate broth, indicating that the contamination did not eliminate these important functional groups of bacteria, in accord with our previous research [13].

Although the use of nitrate broth containing high levels of proteinaceous components is biased towards the enrichment of certain genera, strains belonging to these genera, have been traditionally used for studies of denitrification, and the PCR primers were developed using strains from these commonly cultured genera. Therefore, the primer sets were expected to detect these groups and did detect the control strains. However, PCR amplification products were not produced for many of these strains using NR or DN functional gene primers, or if products were produced, they were not able to be verified by sequencing.

### 4.1. 16S rRNA Phylogeny and the Reduction of Nitrate/Nitrite

The 16S rRNA sequence results showed that nitrate/nitrite-reducing strains were well-represented among γ-Proteobacteria especially the genera of *Stenotrophomonas* (nitrate reducer) and *Pseudomonas* (nitrate/nitrite reducer). Our results were consistent with previous studies regarding the presence of certain groups of NR and DN bacteria in oil/brine-contaminated soils. In different culture-based studies on the composition of NR bacteria communities, *Bacillus*, *Moraxella*, and *Pseudomonas* were isolated from the sediment samples from the rhizosphere of the aerenchymatous plant Glyceria maxima [64]. A larger diversity was observed when Streptomyces, Bacillus, and Enterobacteriaceae strains were isolated from abandoned and reclaimed mine soils [65]. However, a different study showed that DN bacteria were well-represented among members of the genus *Bacillus* under anaerobic conditions [66]. Our 16S rRNA and denitrification genes sequence analysis were consistent in terms of phylogenetic affiliations. Taxonomic identification based on 16S rRNA gene sequences and *nosZ* phylogenetic relationships were also in good agreement for most of the strains studied by Rich et al. (2003) [67] and by Dandie et al. (2007a) [68]. The extensive phylogenetic analysis of multiple denitrification genes performed by Jones et al. (2008) [69] concluded that gene duplication and processes other than horizontal gene transfer were significant factors in the evolution of these genes.

### 4.2. Considerations When Estimating Numbers of NR and DN Bacteria by PCR

Our results show that the simple microtiter plate assay we used is a valuable complement to methods using denitrifier MPN tubes [65] and to PCR-based methods in detecting culturable NR and DN bacteria. With the microtiter plate assay, 54 strains out of 75 were detected as NR or DN bacteria (Table 2), while just ten strains out of the 54 were confirmed to possess the correct functional genes for nitrate reduction or denitrification after sequencing the PCR product, translating the codons into amino acids, and subjecting the translated sequence to BlastP analysis (Supplemental Table S3, Table 3). Therefore, although primers for nitrate reduction and denitrification genes produced PCR products from a number of strains, many of those products could not be confirmed as the expected sequence after analysis, suggesting caution when these primers are used to estimate population sizes of denitrifying bacteria [70,71].

The lack of amplification of NR and DN gene fragments from strains known to be capable of nitrate reduction or denitrification has been previously reported [68,69,72,73], suggesting that reliance on PCR amplification to determine population sizes could lead to underestimates. In addition, reliance on a limited number of genes and primers may cause some important pathways, such as DNRA, to be overlooked [16]. In our study, 44 strains reduced nitrate/nitrite when grown in nitrate broth in the microtiter plate assay, although no PCR products were produced using primers for nitrate/nitrite reductase genes (Supplementary Table S1) in these 44 strains (Supplementary Table S3). Our results suggest that the primers used in this study likely were not perfectly complementary to the nitrate/nitrite reductase genes in these 44 strains.

Our results show that all four isolates (e.g., I-1, I-2, I-4, and I-65) that were confirmed to possess one or more of the denitrification genes showed positive results (e.g., DN positive) in the microtiter plate assay (Table 3). Agreement between the detected requisite genes and the predicted phenotype is not always the case. Two strains of *Bosea* were confirmed to possess gene sequences with high similarity to *napA*, although they did not reduce nitrate in the microtiter plate assay (Table 3, Supplementary Table S4). However, Dandie et al. (2007a) [68] were able to isolate denitrifying *Bosea* strains after enrichment using denitrifier MPN tubes. With respect to denitrification, even with having more than one gene in the denitrification pathway, microorganisms may still lack the ability to denitrify under certain conditions due to their inability to express the genes they possess because those genes must be transcribed and translated into the denitrification enzymes and these enzymes must be active under the given environmental conditions [11].

Estimating population sizes of “denitrifying” bacteria by qPCR of single denitrification genes is complicated by cases in which microbes possess an incomplete denitrification pathway (e.g., the absence of one or more denitrification genes in the denitrification pathway) and, as a result, denitrification stops before the production of nitrogen gas [70,74,75,76]. Conversely, a substantial proportion of denitrifiers may lack *nosZ* [69], which would confound predictions of denitrification rates and N_2_O emissions if population estimates were based on other genes in the denitrification pathway [70]. On the other hand, there is a wide variety of bacteria that possess *nosZ* and reduce N_2_O yet do not contain any other denitrification pathway genes [74]. As noted by Mackelprang et al. (2018) [77], these atypical or “Clade II” *nosZ* genes accounted for the majority of *nosZ* sequences found in a metagenome survey of native prairie soil. In conclusion, we need to know much more about the suites of nitrogen cycling genes carried and expressed by a variety of bacteria before we can confidently use the qPCR of single genes to estimate population sizes of denitrifying bacteria and their role in nitrogen transformations.

### 4.3. Long-Term Impact of Brine/Oil Contamination on Nitrogen Cycling in Tallgrass Prairie Soil

Our results support the hypothesis that the species composition of NR/DN bacteria in brine and oil contamination continued to be affected years after the contamination event. In particular, strains of some groups (e.g., Bacilli) that are known to be salt tolerant [5,78] were found in brine/oil-contaminated soils but not in crude oil-contaminated soils, while most NR/DN strains isolated from the oil-contaminated sites were Gammaproteobacteria. Although it has been reported that many hydrocarbonoclastic bacteria responsible for the first steps of hydrocarbon degradation are classified as γ-Proteobacteria [79], it is not known whether hydrocarbons represent a readily usable carbon source for our NR and DN γ-Proteobacteria strains. Our previous studies of bioremediation at these sites did document the substantial loss of hydrocarbons in association with higher numbers of hydrocarbon-degrading bacteria [34] and of γ-Proteobacteria [37]; however, the hydrocarbon-degrading bacteria were not identified in those studies.

Other long-term effects from previous research indicated that sites contaminated 5–10 years prior had at least ten times the level of nitrate as nearby uncontaminated prairie sampled in parallel [13] despite our findings that NR and DN bacteria were not eliminated by brine/oil contamination. Although certain groups were relatively more or less abundant in oil or brine sites than in the parallel prairie sites, it is not known to what extent this shift in species composition directly affected the rate of nitrogen transformations in tallgrass prairie soil. There were a number of differences between contaminated and uncontaminated sites in terms of environmental parameters, such as soil moisture potentially impacting the activity of nitrate-reducing and denitrifying bacteria. In addition, plant community composition, nematode community structure, and the overall microbial community composition were affected as well [12,13,34,37]. Dominant groups of bacteria in undisturbed TPP included Actinobacteria and Acidobacteria as determined by 16S rRNA sequence analysis but were relatively low in Gammaproteobacteria in contrast to soil contaminated with crude oil [37], which contained a higher proportion of Gammaproteobacteria and lower proportions of Actinobacteria and Acidobacteria. For primarily brine-impacted sites, there was initially about half the PLFA concentration (phospholipid fatty acids, an indication of live cell biomass, [37]) and differences in PFLA structural groups (which roughly correspond to different higher-level taxonomic groups) compared to samples from native prairie. These differences had decreased but not vanished after four years [36,37]. As with oil-contaminated soil, 16S rRNA sequence analysis of brine-contaminated soil had a lower proportion of Acidobacteria and Actinobacteria but higher proportions of Firmicutes, Gammaproteobacteria, and Bacteroidetes [37]. It is noteworthy that at the time of the current study, as much as a decade post-contamination, native prairie had approximately 1/10 the concentration of nitrate as did adjacent contaminated sites, which had never been remediated [13]. Future research should investigate if an alteration in the rate of nitrogen transformations via nitrate reduction and denitrification has a long-term impact on contamination by crude oil and brine.

## 5. Conclusions

We were able to readily isolate and identify 75 strains of bacteria from tallgrass prairie soil contaminated up to 10 years prior with crude oil, a combination of brine and crude oil, or adjacent uncontaminated sites. Fifty-four of these strains proved capable of reducing nitrate and/or nitrite. The strains were affiliated with a variety of different taxonomic groups, with some indication of selection for halotolerant taxa in the primarily brine-contaminated soils and of Gammaproteobacteria in the oil-contaminated soils. However, around 82% of strains shown to reduce nitrate/nitrite would not be identified as NR or DN bacteria by the battery of the functional NR and DN PCR primers used. This low % detection by currently used PCR primers suggests caution in inferring numbers of NR/DN based on PCR amplification. It also attests to the value of a culture-first approach, in which a variety of bacteria are first screened for the ability to reduce nitrate /nitrite, followed by molecular analyses of their functional genes.

## Figures and Tables

**Table 1:** Summary of the 75 strains by genus, origin, and capacity to reduce nitrate/nitrite.

Taxonomic Group	Genus * (Total # of Strains)	Brine/Oil	Oil	Prairie
γ-Proteobacteria	*Stenotrophomonas* (27)	3NR **	2NR, 3“None”	11NR, 1DN 7 “None”
	*Pseudomonas* (9)	2NR, 2DN	1NR	3NR, 1DN
	*Aeromonas* (1)	1NR	0	0
	*Serratia* (1)	0	1NR	0
	*Enterobacter* (3)	0	3NR	0
	*Acinetobacter* (5)	0	0	2NR, 3 “None”
	Total # γ Proteobacteria (46)	8	10	28
α-Proteobacteria	*Ensifer* (2)	1 DN	0	1 DN
	*Bosea* (2)	2 “None”	0	0
	*Phenylobacterium* (1)	1NR	0	0
	Total # α- Proteobacteria (5)	4	0	1
β-Proteobacteria	*Achromobacter* (4)	1NR	0	3NR
	*Burkholderia* (1)	0	0	1 “None”
	Total # β- Proteobacteria (5)	1	0	4
Actinobacteria	*Arthrobacter* (1)	1 “None”	0	0
	*Rhodococcus* (1)	0	0	1 “None”
	*Microbacterium* (1)	0	1NR	0
	*Kocuria* (1)	0	0	1NR
	Total # Actinobacteria (4)	1	1	2
Bacilli	*Brevibacillus* (8)	3NR	0	4NR, 1“None”
	*Bacillus* (3)	2NR	0	1”None”
	*Paenibacillus* (1)	1NR	0	0
	*Lysinibacillus* (2)	0	0	1NR, 1”None”
	*Total # Bacilli* (14)	6	0	8
Flavobacteria	*Chryseobacterium* (1)	0	1NR	0
	Total # Flavobacteria (1)	0	1	0
	Total # strains isolated	20	12	43
	Total # NR + DN	17	9	28
	#NR, #DN	14, 3	9, 0	25, 3

*: Genus assignments as determined by submitting the 16S rRNA gene sequence of the individual strains to The Ribosomal Database Project (RDP) Classifier. All classifications were at the 98% threshold level or higher. Origin: type of site from which the strain was isolated. See Supplementary Table S2. Brine/oil: isolated from sites contaminated primarily by oil field brine but also by crude oil, G5, and G7. Oil: isolated from sites contaminated by crude oil, J6-F, J6-NF, and LF. Prairie: isolated from uncontaminated sites, G5P, G7P, J6P, and LFP. **: number and type of strain. Strain type: NR, DN, None: confirmed as NR or DN by reduction of nitrate to nitrite, or no nitrate or nitrite detected (“DN”) after growth in nitrate broth and testing with Griess reagents. Strains that did not reduce nitrate were designated as “None”. See materials and methods for more information.

**Table 2:** Summary of the 75 strains by genus, nitrate/nitrite reduction, and detection of molecular markers.

Taxonomic Group	Genus * (Total # of Strains, # Screened for *qnorB*, c*norB, nosZ*)	NR ^a^	DN ^b^	None ^c^
γ-Proteobacteria	*Stenotrophomonas* (27, 15)	16 *narG* (2)	1	10
	*Pseudomonas* (9, 7)	6 *napA* (2)	3 *napA* (1), *nirS* (2), *cnorB* (1), *nosZ* (1)	0
	*Aeromonas* (1, 1)	1	0	0
	*Serratia* (1, 1)	1	0	0
	*Enterobacter* (3, 1)	3	0	0
	*Acinetobacter* (5, 1)	2	0	3
α-Proteobacteria	*Ensifer* (2, 1)	0	2 *napA* (1), *cnorB* (1)	0
	*Bosea* (2, 0)	0	0	2 *napA* (2)
	*Phenylobacterium* (1, 1)	1	0	0
β-Proteobacteria	*Achromobacter * (4, 2)	4 *napA* (1)	0	0
	*Burkholderia* (1, 0)	0	0	1
Actinobacteria	*Arthrobacter* (1, 0)	0	0	1
	*Rhodococcus* (1, 0)	0	0	1
	*Microbacterium* (1, 1)	1	0	0
	*Kocuria* (1, 1)	1	0	0
Bacilli	*Brevibacillus* (8, 1)	7	0	1
	*Bacillus* (3, 0)	2	0	1
	*Paenibacillus* (1, 0)	1	0	0
	*Lysinibacillus* (2, 0)	1	0	1
Flavobacteria	*Chryseobacterium* (1, 0)	1	0	0
Total	75 (33)	48	6	21

Strains were identified from their 16S rRNA gene sequences and their ability to reduce nitrate and/or nitrite by the microtiter plate assay as described. All 75 strains were screened using the primer sets for *napA*, *narG*, *nirS,* and *nirK* (Supplementary Table S1). A subset of 33 strains were screened using the primer sets for *qnorB*, *cnorB*, *qnorB*, and *nosZ*. *: Genus assignments were determined by submitting the 16S rRNA gene sequence of the individual strains to The Ribosomal Database Project (RDP) Classifier. ^a^ Nitrate Reducers. ^b^ Nitrite Reducers. ^c^ None: no reduction of nitrate or nitrite.

**Table 3:** Strains in which gene sequences for nitrate reduction or denitrification were detected.

Strain # (Genus)	Soil Type	*napA*	*narG*	*nirS*	*cnorB*	*nosZ*	NR or DN *
1 (*Pseudomonas*)	Brine/Oil	Yes		Yes			DN
2 (*Pseudomonas*)	Brine/Oil			Yes			DN
4 (*Ensifer*)	Brine/Oil				Yes		DN
7 (*Bosea*)	Brine/Oil	Yes					None
8 (*Bosea*)	Brine/Oil	Yes					None
39 (*Stenotrophomonas*)	Prairie		Yes				NR
42 (*Stenotrophomonas*)	Prairie		Yes				NR
49 (*Achromobacter*)	Prairie	Yes					NR
65 (*Pseudomonas*)	Prairie				Yes	Yes	DN
67 (*Pseudomonas*)	Prairie	Yes					NR
68 (*Pseudomonas*)	Prairie	Yes					NR
69 (*Ensifer*)	Prairie	Yes					NR

NR: Nitrate Reduction. DN: Nitrite reduction/denitrification. Genus: 16S rRNA sequence as classified by The RDP Classifier. * Loss of nitrate (NR) or nitrite (DN) after incubation in nitrate broth. *napA*: periplasmic nitrate reductase. *narG*: membrane-bound nitrate reductase. *nirS*: Cytochrome cd1-nitrite reductase. *cnorB*: Nitric oxide reductase gene. *nosZ*: nitrous oxide reductase gene. No verified PCR product was obtained for *nirK* (Copper nitrite reductase) or for *qnorB* (Nitric oxide reductase gene).

## Data Availability

The raw data supporting the conclusions of this article will be made available by the authors on request.

## Supplementary Materials

The following supporting information can be downloaded at https://www.mdpi.com/article/10.3390/microorganisms12101981/s1, Table S1: Summary of primers used to detect denitrification functional genes; Table S2: Site, site type, dilution origin, and phenotype, for 75 strains; Table S3: Genbank accession numbers for 16S rRNA gene sequence and functional genes for nitrate reducing and denitrifying genes; Table S4: Summary of nitrate reducing/denitrification genes from isolates (Accession number “in bold”/Closest GenBank match accession # (% similarity)).
