# Supplementary material for: Detection of Nitrate-Reducing/Denitrifying Bacteria from Contaminated and Uncontaminated Tallgrass Prairie Soil: Limitations of PCR Primers
Source: Microorganisms. 2024 Sep 30;12(10):1981. doi: 10.3390/microorganisms12101981 (PMC11509419; doi:10.3390/microorganisms12101981)
Supplement: Supplementary file 1 [file microorganisms-12-01981-s001.zip › microorganisms-3202100-supplementary.pdf]

Supplemental Table S1. Summary of primers used to detect denitrification functional genes.

Primer[a]	Primer sequence (5'-3')	Organism[b]	Size of the PCR fragment (bp)	Annealing temperature (˚C)	Reference
napA-F1	CTGGACIATGGGYTTIAACCA	*Escherichia coli* O6	492	52˚C	46
napA-R1	CCTTCYTTYTCIACCCACAT				
narG-F1	ACICAYGGIGTIAACTGYAC	*Pseudomonas stutzeri* ATCC 17588	523	52˚C	46
narG-R1	TCGSMRTACCAGTCRTARAA				
nirS 1F	CCTAYTGGCCGCCRCART	*Pseudomonas aeruginosa* ATCC 27853	890	Touchdown 56-51˚C	28
nirS 6R	CGTTGAACTTRCCGGT				
nirK F	TCATGGTCCTGCCGCGYGACGG	*Alcaligenes faecalis* ATCC 8750	329	Touchdown 63-53˚C	32
nirK R	GAACTTGCCGGTNGCCCAGAC				
cnorB 2F	GACAAGNNNTACTGGTGGT	*Pseudomonas aeruginosa* ATCC 27853	389	Touchdown 57-52.5˚C	26
cnorB 6R	GAANCCCCANACNCCNGC				
qnorB 2F	GGNCAYCARGGNTAYGA	*Alcaligenes faecalis* ATCC 8750	637	Touchdown 57-52.5˚C	26
qnorB 7R	GGNGGRTTDATCADGAANCC				
nosZ-F-1181	CGCTGTTCITCGACAGYCAG	*Pseudomonas stutzeri* ATCC 17588	680	56˚C	67
nosZ-R-1880	ATGTGCAKIGCRTGGCAGAA				

[a]Forward and reverse primers are indicated by F and R as the last letter, respectively.

[b] Strains used for PCR positive control.

Supplemental Table S2. Site, site type, dilution origin, and phenotype, for 75 strains.

Strain #	Site	Site type	Dilution Origin[a]	Phenotype[b]
1	G7	Brine/oil	10^{-3}	DN
2	G7	Brine/oil	10^{-3}	DN
3	G7	Brine/oil	10^{-3}	NR
**4**	**G7**	**Brine/oil**	$\mathbf{10^{-4}}$	**DN**
5	G7	Brine/oil	10^{-3}	NR
6	G7	Brine/oil	10^{-3}	NR
**7**	**G7**	**Brine/oil**	$\mathbf{10^{-4}}$	**None**
**8**	**G7**	**Brine/oil**	$\mathbf{10^{-4}}$	**None**
**9**	**G7**	**Brine/oil**	$\mathbf{10^{-5}}$	**None**
**10**	**G7**	**Brine/oil**	$\mathbf{10^{-5}}$	**NR**
11	G5	Brine/oil	10^{-2}	NR
12	G5	Brine/oil	10^{-3}	NR
**13**	**G5**	**Brine/oil**	$\mathbf{10^{-4}}$	**NR**
14	G5	Brine/oil	10^{-2}	NR
15	G5	Brine/oil	10^{-3}	NR
16	G5	Brine/oil	10^{-2}	NR
17	G5	Brine/oil	10^{-2}	NR
18	G5	Brine/oil	10^{-2}	NR
19	G5	Brine/oil	10^{-2}	NR
20	G5	Brine/oil	10^{-2}	NR
21	LF	Oil	10^{-3}	NR
22	LF	Oil	10^{-3}	NR
23	LF	Oil	10^{-3}	NR
**24**	**LF**	**Oil**	$\mathbf{10^{-4}}$	**NR**
**25**	**LF**	**Oil**	$\mathbf{10^{-5}}$	**NR**
26	J6-NF	Oil	10^{-2}	None
27	J6-NF	Oil	10^{-3}	None
**28**	**J6-NF**	**Oil**	$\mathbf{10^{-4}}$	**None**
29	J6-F	Oil	10^{-2}	NR
30	J6-F	Oil	10^{-2}	NR
31	J6-F	Oil	10^{-3}	NR
32	J6-F	Oil	10^{-3}	NR
33	G5P	Prairie	10^{-2}	NR
34	G5P	Prairie	10^{-2}	NR
35	G5P	Prairie	10^{-2}	NR
36	G5P	Prairie	10^{-2}	NR
37	G5P	Prairie	10^{-2}	NR
38	G5P	Prairie	10^{-2}	NR
39	G5P	Prairie	10^{-2}	NR
40	G5P	Prairie	10^{-2}	NR
41	G5P	Prairie	10^{-3}	NR
**42**	**G5P**	**Prairie**	$\mathbf{10^{-4}}$	**NR**
43	J6P	Prairie	10^{-2}	NR
44	J6P	Prairie	10^{-2}	None
45	J6P	Prairie	10^{-2}	NR

46	J6P	Prairie	10^{-3}	NR
47	J6P	Prairie	10^{-3}	None
**48**	**J6P**	**Prairie**	**10^{-4}**	**None**
49	G7P	Prairie	10^{-2}	NR
50	G7P	Prairie	10^{-2}	None
51	G7P	Prairie	10^{-2}	None
52	G7P	Prairie	10^{-2}	None
**53**	**G7P**	**Prairie**	**10^{-4}**	**NR**
54	G7P	Prairie	10^{-3}	NR
**55**	**G7P**	**Prairie**	**10^{-4}**	**None**
**56**	**G7P**	**Prairie**	**10^{-4}**	**NR**
57	G7P	Prairie	10^{-2}	DN
58	G7P	Prairie	10^{-2}	None
59	G7P	Prairie	10^{-3}	NR
60	G7P	Prairie	10^{-3}	None
61	LFP	Prairie	10^{-2}	NR
62	LFP	Prairie	10^{-2}	None
63	LFP	Prairie	10^{-2}	None
64	LFP	Prairie	10^{-2}	None
65	LFP	Prairie	10^{-3}	DN
66	LFP	Prairie	10^{-3}	None
**67**	**LFP**	**Prairie**	**10^{-4}**	**NR**
**68**	**LFP**	**Prairie**	**10^{-4}**	**NR**
69	LFP	Prairie	10^{-2}	DN
70	LFP	Prairie	10^{-2}	NR
71	LFP	Prairie	10^{-3}	None
72	LFP	Prairie	10^{-3}	None
**73**	**LFP**	**Prairie**	**10^{-4}**	**NR**
74	LFP	Prairie	10^{-2}	NR
75	LFP	Prairie	10^{-3}	NR

*The highly abundant strains (e.g. originated from wells with 10^{-4} or 10^{-5} dilution) are indicated in bold letters.

Site Type:

Brine/oil: contaminated by a spill containing both oil-field brine (salt water) and crude oil (G5 and G7), at approximately 10:1 brine to oil ratio (vol/vol).

Oil: contaminated by a spill of crude oil (LF, J6-NF and J6-F).

Prairie: Uncontaminated (G5P, G7P, J6P, LFP)

[a] Dilution corresponded to microtiter plate-wells from which strains were originated, e.g. 10^{-3} corresponds to a 1:1000 final dilution of soil: saline dilutant into nitrate broth. See materials and methods for more information

[b] Phenotype: NR: nitrate reduction, DN: nitrite reduction/denitrification, None: no reduction of nitrate or nitrite.

Supplemental Table S3. Genbank accession numbers for 16S rRNA gene sequence and functional genes for nitrate reducing and denitrifying genes.

Isolate #/ Phenotype (e.g. NR, DN, or None)	Isolate name (16S rRNA Accession #)	Genus [% similarity][1] (Class/subclass)	Closest GenBank match accession # (% similarity)	*napA*	*narG*	*nirS*	*nirK*	*cnorB*	*nosZ*
**1 DN**	**G7-101B3B (JQ917765)**	***Pseudomonas* [100%] (γ)**	**KU977117 (99.86%)**	++*	+	++*		+	+
**2 DN**	**G7-105B12A (JQ917766)**	***Pseudomonas* [100%] (γ)**	**MG754443 (100%)**	+	+	++*		+	+
3 NR	G7-129B8B (JQ917767)	*Pseudomonas* [100%] (γ)	MN208153 (99.57%)						
**4 DN**	**G7-135C4 (JQ917768)**	***Ensifer* [99%] (α)**	**KU877644 (100%)**					++*	
**5 NR**	**G7-221B10A (JQ917769)**	***Achromobacter* [99%] (β)**	**MK789752 (99.86%)**		+				+
**6 NR**	**G7-221B10B (JQ917770)**	***Aeromonas* [100%] (γ)**	**KJ157321 (100%)**	+					+
7 None	G7-305C11A1 (JQ917771)	*Bosea* [100%] (α)	MG775032 (99.85%)	++*					
8 None	G7-305C11B (JQ917772)	*Bosea* [100%] (α)	ON698112 (99.71%)	++*					
9 None	G7-325D2 (JQ917773)	*Arthrobacter* [100%] (Actino)	KM817229 (99.93%)						
**10 NR**	**G7-401D6 (JQ917774)**	***Phenylobacterium* [100%] (α)**	**FJ605405 (99.93%)**						
**11 NR**	**G5NA3A (JQ917775)**	***Stenotrophomonas* [100%] (γ)**	**MK475029 (99.71%)**						

12<br>NR	G5NB3B<br>(JQ917776)	*Pseudomonas*<br>[95%] (γ)	MH703445<br>(98.83%)	+					
**13<br>NR**	**G5NC6A2<br>(JQ917777)**	***Stenotrophomonas***<br>**[100%] (γ)**	**KY820657<br>(99.93%)**						
14<br>NR	G5MA8<br>(JQ917778)	*Brevibacillus*<br>[100%] (Bacilli)	MW736872<br>(99.49%)						
15<br>NR	G5MB8A<br>(JQ917779)	*Bacillus*<br>[100%] (Bacilli)	GU568201<br>(99.93%)						
16<br>NR	G5MA9A2<br>(JQ917780)	*Brevibacillus*<br>[100%] (Bacilli)	KT363753<br>(99.63%)						
17<br>NR	G5MA10B<br>(JQ917781)	*Paenibacillus*<br>[100%] (Bacilli)	KT719740<br>(99.35%)						
**18<br>NR**	**G5SA2A1<br>(JQ917782)**	***Stenotrophomonas***<br>**[100%] (γ)**	**ON556407<br>(99.79%)**		+				
19<br>NR	G5SA3A<br>(JQ917783)	*Brevibacillus*<br>[100%] (Bacilli)	MT427642<br>(99.64%)						
20<br>NR	G5SA5A<br>(JQ917784)	*Bacillus*<br>[100%] (Bacilli)	MK592620<br>(99.42%)						
**21<br>NR**	**LF-521B12<br>(JQ917785)**	***Stenotrophomonas***<br>**[100%] (γ)**	**JN867123<br>(100%)**						
22<br>NR	LF-<br>617B2A1A<br>(JQ917786)	*Chryseobacterium*<br>[100%] (Flavo)	JN208181<br>(99.71%)						
**23<br>NR**	**LF-<br>617B2A1B<br>(JQ917787)**	***Stenotrophomonas***<br>**[100%] (γ)**	**HQ670707<br>(99.29%)**						
**24<br>NR**	**LF-<br>821C8A1<br>(JQ917788)**	***Serratia***<br>**[100%] (γ)**	**MT538443<br>(99.93%)**		+				
**25<br>NR**	**LF-825D4<br>(JQ917789)**	***Enterobacter***<br>**[99%] (γ)**	**HM854373<br>(99.86%)**		+		+		

26<br>None	J6N-NFA4A<br>(JQ917790)	*Stenotrophomonas*<br>[100%] (γ)	MG905299<br>(99.28%)						
27<br>None	J6N-<br>NFB4B1<br>(JQ917791)	*Stenotrophomonas*<br>[100%] (γ)	MN181024<br>(99.14%)						
28<br>None	J6N-<br>NFC6A2<br>(JQ917792)	*Stenotrophomonas*<br>[100%] (γ)	MG905288<br>(99.93%)		+				
**29**<br>**NR**	**J6N-FA10A**<br>**(JQ917793)**	***Microbacterium***<br>**[100%] (Actino)**	**MH168999**<br>**(100%)**						
**30**<br>**NR**	**J6N-FA10C**<br>**(JQ917794)**	***Pseudomonas***<br>**[100%] (γ)**	**AB621834**<br>**(100%)**						
31<br>NR	J6N-FB11B<br>(JQ917795)	*Enterobacter*<br>[92%] (γ)	MT102735<br>(99.27%)		+				
32<br>NR	J6N-FB11C<br>(JQ917796)	*Enterobacter*<br>[100%] (γ)	MN181146<br>(99.64%)		+				
33<br>NR	G5PA8B1<br>(JQ917797)	*Lysinibacillus*<br>[100%] (Bacilli)	MT507231<br>(99.86%)						
**34**<br>**NR**	**G5PA8B2**<br>**(JQ917798)**	***Stenotrophomonas***<br>**[100%] (γ)**	**KY849353**<br>**(99.21%)**		+				
**35**<br>**NR**	**G5PA9A1**<br>**(JQ917799)**	***Stenotrophomonas***<br>**[100%] (γ)**	**MT543133**<br>**(99.50%)**		+				
**36**<br>**NR**	**G5PA9A2**<br>**(JQ917800)**	***Stenotrophomonas***<br>**[100%] (γ)**	**MH429813**<br>**(99.86%)**						
**37**<br>**NR**	**G5PA9B**<br>**(JQ917801)**	***Stenotrophomonas***<br>**[100%] (γ)**	**MW695215**<br>**(99.86%)**		+				+
**38**<br>**NR**	**G5PA10A1**<br>**(JQ917802)**	***Stenotrophomonas***<br>**[100%] (γ)**	**HQ185400**<br>**(99.64%)**		+				
**39**<br>**NR**	**G5PA10A2**<br>**(JQ917803)**	***Stenotrophomonas***<br>**[100%] (γ)**	**MN372320**<br>**(99.64%)**		++*				
**40**<br>**NR**	**G5PA10B**<br>**(JQ917804)**	***Stenotrophomonas***<br>**[100%] (γ)**	**ON357999**<br>**(99.36%)**		+				

**41**<br>**NR**	**G5PB12A1**<br>**(JQ917805)**	***Stenotrophomonas***<br>**[100%] (γ)**	**ON357998**<br>**(99.43%)**		+				
**42**<br>**NR**	**G5PC11**<br>**(JQ917806)**	***Stenotrophomonas***<br>**[100%] (γ)**	**MH788995**<br>**(99.28%)**		++*				
43<br>NR	J6PA9A<br>(JQ917807)	*Achromobacter*<br>[100%] (β)	ON831558<br>(99.78%)						
44<br>None	J6PA9B2<br>(JQ917808)	*Lysinibacillus*<br>[100%] (Bacilli)	MK789730<br>(99.37%)						
45<br>NR	J6PA12A<br>(JQ917809)	*Achromobacter*<br>[100%] (β)	MH773230<br>(95.68%)						
46<br>NR	J6PB8A<br>(JQ917810)	*Brevibacillus*<br>[100%] (Bacilli)	KU500374<br>(99.56%)						
47<br>None	J6PB8B<br>(JQ917811)	*Brevibacillus*<br>[100%] (Bacilli)	MW736860<br>(99.79%)						
48<br>None	J6PC12A<br>(JQ917812)	*Rhodococcus*<br>[100%] (Actino)	KT380589<br>(97.65%)						
**49**<br>**NR**	**G7P1A3A1**<br>**(JQ917813)**	***Achromobacter***<br>**[100%] (β)**	**KY206819**<br>**(99.93%)**	++*	+		+		+
50<br>None	G7P1A3A2<br>(JQ917814)	*Stenotrophomonas*<br>[100%] (γ)	OQ968958<br>(99.43%)						
51<br>None	G7P1A3B1<br>(JQ917815)	*Stenotrophomonas*<br>[100%] (γ)	ON448385<br>(99.28%)						
52<br>None	G7P1A3B2<br>(JQ917816)	*Stenotrophomonas*<br>[100%] (γ)	KU551125<br>(99.86%)						
**53**<br>**NR**	**G7P1C4**<br>**(JQ917817)**	***Brevibacillus***<br>**[100%]** (Bacilli)	**KX170837**<br>**(99.93%)**						
54<br>NR	G7P1B2<br>(JQ917818)	*Brevibacillus*<br>[100%] (Bacilli)	MT052647<br>(99.55%)						
55<br>None	G7P2C6A<br>(JQ917819)	*Acinetobacter*<br>[100%] (γ)	MH671640<br>(98.90%)						
56<br>NR	G7P2C6B<br>(JQ917820)	*Acinetobacter*<br>[100%] (γ)	MN733120<br>(100%)						

57<br>DN	G7P2A3A<br>(JQ917821)	*Stenotrophomonas*<br>[100%] (γ)	KF737383<br>(99.49%)						
58<br>None	G7P2A3B<br>(JQ917822)	*Acinetobacter*<br>[100%] (γ)	MH130300<br>(98.04%)						
59<br>NR	G7P2B3A<br>(JQ917823)	*Brevibacillus*<br>[100%] (Bacilli)	KT363755<br>(99.64%)						
60<br>None	G7P2B3B<br>(JQ917824)	*Bacillus*<br>[100%] (Bacilli)	MT605503<br>(99.90%)		+				
**61**<br>**NR**	**LFP1A9A1**<br>**(JQ917825)**	***Stenotrophomonas***<br>**[100%] (γ)**	**LC506130**<br>**(100%)**						
62<br>None	LFP1A9A2<br>(JQ917826)	*Stenotrophomonas*<br>[100%] (γ)	MG833394<br>(99.50%)						
63<br>None	LFP1A9B1<br>(JQ917827)	*Stenotrophomonas*<br>[100%] (γ)	MK612128<br>(99.43%)						
64<br>None	LFP1A9B2<br>(JQ917828)	*Stenotrophomonas*<br>[100%] (γ)	KT580582<br>(99.93%)						
**65**<br>**DN**	**LFP1B10A**<br>**(JQ917829)**	***Pseudomonas***<br>**[100%] (γ)**	**KC207086**<br>**(99.93%)**		+			++*	++*
66<br>None	LFP1B10B<br>(JQ917830)	*Burkholderia*<br>[100%] (β)	KF788042<br>(99.35%)						
**67**<br>**NR**	**LFP1C12A**<br>**(JQ917831)**	***Pseudomonas***<br>**[100%] (γ)**	AY623928<br>**(99.93%)**	++*					
**68**<br>**NR**	**LFP1C12B**<br>**(JQ917832)**	***Pseudomonas***<br>**[100%] (γ)**	**OR358908**<br>**(99.50%)**	++*					
69<br>DN	LFP2A8A2<br>(JQ917833)	*Ensifer*<br>[100%] (α)	MT431909<br>(100%)	++*					
**70**<br>**NR**	**LFP2A8B**<br>**(JQ917834)**	***Acinetobacter***<br>**[100%] (γ)**	**MN696228**<br>**(100%)**						
71<br>None	LFP2B10A<br>(JQ917835)	*Stenotrophomonas*<br>[100%] (γ)	MN889390<br>(99.43%)						
72<br>None	LFP2B10B1<br>(JQ917836)	*Acinetobacter*<br>[100%] (γ)	MN082060<br>(100%)						

**73**<br><br>**NR**	**LFP2C11**<br><br>**(JQ917837)**	***Pseudomonas***<br><br>**[100%] (γ)**	**MT539779 (99.86%)**	+					
74<br><br>NR	LFP2A8A1<br><br>(JQ917838)	*Stenotrophomonas*<br><br>[100%] (γ)	JQ675547 (99.93%)						
**75**<br><br>**NR**	**LFP2B10B2**<br><br>**(JQ917839)**	***Kocuria***<br><br>**[100%] (Actino)**	**MK205170 (100%)**						

Isolate name: internal description of strain.

[1]Threshold level of similarity as determined by The Ribosomal Database Project (RDP) Classifier program.

NR: nitrate reduction.

DN: nitrite reduction/denitrification.

None: no reduction of nitrate or nitrite.

+: PCR amplification product obtained

++*: Sequencing of PCR product confirmed identity

The 33 selected strains are indicated in bold letters.

napA: periplasmic nitrate reductase.

narG: membrane-bound nitrate reductase.

nirS: cytochrome cd1-nitrite reductase.

nirK: copper nitrite reductase

cnorB: cytochrome bc-type complex nitric oxide reductase gene.

nosZ: nitrous oxide reductase gene.

Note: no amplification products were obtained using the *qnorB* primers.

Supplemental Table S4. Summary of nitrate reducing/denitrification genes from isolates (Accession number "in bold"/ Closest GenBank match accession # (% similarity))

Isolate #/<br>NR/DN	*napA*/<br>Accession #	*narG*/<br>Accession #	*nirS*/<br>Accession #	*cnorB*/<br>Accession #	*nosZ*/<br>Accession #
1<br>DN	**OR416962**/<br>*Pseudomonas frederiksbergensis* strain AS1<br>CP018319,<br>98%		**OR416971**/<br>*Pseudomonas frederiksbergensis* strain AS1<br>CP018319,<br>99%		
2<br>DN			**OR416972**/<br>*Pseudomonas frederiksbergensis* strain AS1<br>CP018319,<br>98%		
4<br>DN				**OR416960** *Ensifer adhaerens* strain NER9<br>CP101518,<br>96%	
7<br>None	**OR416963**/<br>*Bosea beijingensis* strain REN20<br>CP132359,<br>91%				
8<br>None	**OR416964**/<br>*Bosea beijingensis* strain REN20<br>CP132359,<br>91%				
39<br>NR		**OR416969**/<br>*Stenotrophomonas geniculata* strain BR23<br>CP134450,<br>99.61%			
42<br>NR		**OR416970**/<br>*Stenotrophomonas geniculata* strain BR23<br>CP134450,<br>99.61%			
49<br>NR	**OR416965**/<br>*Achromobacter pestifer* strain FDAARGOS_79<br>CP053985,<br>95.85%				
65<br>DN				**OR416961**/<br>Pseudomonadaceae bacterium isolate f13e6c2e-50f2-4c75-b4f3-64e691971528 ,<br>OY763427 ,<br>89.21%	**OR416973**/<br>*Pseudomonas* sp. P9_35,<br>CP125373,<br>97.35%

67<br>NR	**OR416966**/<br>*Pseudomonas umsongensis* strain BS3657<br>LT629767, 98%				
68<br>NR	**OR416967**<br>*Pseudomonas umsongensis* strain BS3657<br>LT629767, 98%				
69<br>DN	**OR416968**/<br>*Ensifer adhaerens* strain Corn53<br>CP030262, 98.76%				

NR: nitrate reduction

DN: nitrite reduction/denitrification

None: no reduction of nitrate or nitrite.

*% similarity of isolate sequence to that of closest GenBank match, excluding uncultured or environmental sample sequences.

napA: periplasmic nitrate reductase.

narG: membrane-bound nitrate reductase.

nirS: cytochrome cd1-nitrite reductase.

cnorB: cytochrome bc-type complex nitric oxide reductase gene.

nosZ: nitrous oxide reductase gene.
